# SELF-PERCEPTIONS OF AGING, SUPPORT FROM CLOSE SOCIAL TIES, AND HEALTH GOAL PROGRESS IN EVERYDAY LIFE

**DOI:** 10.1093/geroni/igac059.900

**Published:** 2022-12-20

**Authors:** Shannon Mejía, Karen Hooker

**Affiliations:** University of Illinois at Urbana-Champaign, Champaign, Illinois, United States; Oregon State University, Corvallis, Oregon, United States

## Abstract

Subjective aging is interpersonal, it embodies processes of thinking about the self in relationship to others. We utilize data from the 100-day microlongitudinal Personal Understanding of Life and Social Experiences Project (N = 99; observations = 7,049; Mage = 63, range = 52-88) to explore how self-perceptions of aging differentiate the processes by which older adults shape interactions with close social ties to support progress toward a meaningful health goal in everyday life. Those with more positive self-perceptions of aging identified more friends among their closest social ties and reported a higher levels of goal progress and support toward that goal during the study period. Further, multilevel random coefficient models showed that goal progress on a given day was more sensitive to received support on that day among those with more positive self-perceptions of aging. The implications for adult development and shared experiences of aging within friendship networks is discussed.

